# The constrained-disorder principle defines the functions of systems in nature

**DOI:** 10.3389/fnetp.2024.1361915

**Published:** 2024-12-18

**Authors:** Yaron Ilan

**Affiliations:** Faculty of Medicine, Hadassah Medical Center, Hebrew University, Jerusalem, Israel

**Keywords:** variability, complexity, law of physics, artificial intelligence, disorder, biological noise, complex systems

## Abstract

The Constrained Disorder Principle (CDP) defines all systems in nature by their degree of inherent variability. Per the CDP, the intrinsic variability is mandatory for their proper function and is dynamically changed based on pressures. The CDP defines the boundaries of inherent variability as a mechanism for continuous adaptation to internal and external perturbations, enabling survival and function under dynamic conditions. The laws of nature govern the world’s natural phenomena and underlie the function of all systems. Nevertheless, the laws of physics do not entirely explain systems’ functionality under pressure, which is essential for determining the correct operation of complex systems in nature. Variability and noise are two broad sources of inherent unpredictability in biology and technology. This paper explores how the CDP defines the function of systems and provides examples from various areas in nature where the CDP applies, including climate, genetic, biology, and human behavioral variabilities. According to the CDP, system malfunction results from inappropriate performance of the boundaries of inherent variability. The environment influences the physiological variability, and species interactions influence eco-evolutionary outcomes. The CDP defines human behavior as being driven by randomness and accounts for malfunctions and their corrections. The paper reviews variability-based CDP algorithms and CDP-based second-generation artificial intelligence systems and their potential for improving systems’ prediction and efficiency by using variability.

## 1 Introduction

A law of nature is a rule that governs the world’s natural phenomena. The natural world obeys the laws of nature. Nature’s laws attempt to explain how systems function (Earman et al., 1984). Nevertheless, the laws of physics cannot entirely explain system functionality under pressure, which is essential for elucidating the correct operation of systems (Law of nature, 2024). The Constrained Disorder Principle (CDP) defines all systems in nature by their degree of inherent variability, which is mandatory for their proper function and is dynamically changed based on pressures (Ilan, 2022a).

This paper explores how the CDP defines the function of systems in nature and accounts for malfunctions. It describes the use of CDP-based platforms to improve systems’ functionality. The paper provides examples from various fields, highlighting the concept’s relevance and applicability to various areas.

### 1.1 The current laws of physics are insufficient to explain the functionality of all systems in nature

Classical physics laws have been used to explain phenomena in the macroscopic world. Examples include the classical laws of motion, gravity, electromagnetism, thermodynamics, and energy. These empirically based natural laws govern macroscopic physical systems (Wong et al., 2023). These laws codify nature’s phenomena at human perception scales, focusing on nonrelativistic phenomena greater than quantum effects. Most of these laws can be quantified as equations based on measurable parameters, such as mass, force, acceleration, distance, energy, or charge (Wong et al., 2023).

While the classical laws of physics can explain many observable natural phenomena, they cannot codify natural phenomena alone, even when combined with several assumptions and initial conditions (Broz et al., 2023). Recently, a natural law called the law of increasing functional information was proposed using functional information as a parameter (Wong et al., 2023). However, it may not thoroughly explain how all systems in nature function under dynamic conditions.

### 1.2 The constrained disorder principle defines the function of all systems in nature

The constrained disorder principle (CDP) defines all systems in nature based on their degree of variability. Per the CDP, all systems are characterized by inherent variability, which is mandatory for proper function. The degree of variability is determined by the dynamic borders, which adapt the range of the inherent variability to the internal and external pressures. It provides systems with a mechanism for adaptation under continuous perturbations (Ilan, 2022a). Noise is defined from the point of view of both dynamics and statistics. A CDP is schematically formulated using the B=F formula, where B represents borders and F represents function and efficiency. The formula implies that the boundaries of variability determine the proper function of systems. As a result of this mechanism, systems can better adapt to the noise around them and, therefore, conserve energy (Ilan, 2022b). A malfunction occurs when the borders cannot contain more variability when needed or cannot limit the amount of variability when its degree is too high, disturbing the adequate function (Sigawi et al., 2023).

In a closed system, energy is conserved according to the first law of thermodynamics (Zohuri and Zohuri, 2018). According to the second law of thermodynamics, heat cannot flow spontaneously from colder to warmer bodies, and the entropy of a closed system remains constant or increases (Lieb and Yngvason, 2004; Zivieri, 2023). Based on the second law, the CDP provides a platform that underpins all system functionality, including the microscopic and macroscopic worlds. It enables systems to conserve energy by adapting to noise rather than attempting to repel it (Ilan, 2022b).

### 1.3 Variability and noise are two broad sources of inherent unpredictability in biology and technology

According to the CDP, every system in nature is characterized by noise (Ilan, 2022b; Ilan, 2019a; Ilan, 2019b; Ilan, 2020a; Ilan, 2020b; El-Haj et al., 2019; Ilan, 2019c; Ilan, 2019d; Ilan-Ber and Ilan, 2019; Forkosh et al., 2020; Ilan, 2019e; Ilan, 2022c; Shabat et al., 2021; Ilan, 2023a). Variabilities within and between systems determine the function and outcome of all systems (Ilan, 2023a; Ilan, 2023b). It is thus necessary to dissect intra- and intersystem variability and technical noise to understand functionality and malfunctions.

Replicability is a crucial benchmark for good research. It is often thought that something has gone wrong when results differ from those from a previous study of the same system using the same method (Prasad, 2023). However, many variables contributing to the outcome are often overlooked in an attempt to understand a system. Two broad sources of unpredictability exist when applying the same method to an identical problem: the inherent variability in a system and the noise associated with the technology used (Prasad, 2023).

In addition to the inherent variability of brain function necessary for proper functioning, technical noise and intra and inter-patient variabilities must be considered (Van Horn et al., 2008; Waschke et al., 2021). For example, a technical difference in neuroimaging can result in noise, which depends on the type of MRI scanner, scanning protocols, and software version used for analysis (Prasad, 2023). Different populations have different brain morphology, so normative data and brain templates based on European ancestry are unsuitable for other brain types (Kang et al., 2020). In some areas of the world, genetic variations are more prevalent, affecting the results of brain testing (Kopal et al., 2023). The aging process and the effects of sex have a direct effect on the brain. Globally, population ages vary, affecting participants’ ages and the results of brain imaging (Kapoor et al., 2019). Neuroimaging is influenced by socioeconomic factors that are different across countries (Karia et al., 2021). In studies in which a disease has distinct phenotypes, the proportion of clinical phenotypes included in the research affects the results (Gutiérrez-Gutiérrez et al., 2021). Diseases vary biologically between countries. Parkinson’s disease onset is a decade younger in India than in Western countries (Prasad et al., 2022). It exemplifies the importance of accounting for technical noise and inter-subject variabilities when considering the inherent intra-system variability.

### 1.4 CDP defines the variability of systems in nature

A few examples from natural systems illustrate that inherent intra-system variability is essential to proper system functionality.

#### 1.4.1 The CDP defines climate variability

Climate variability refers to changes in climatic parameters from their long-term mean. The climate of a location varies every year during a specific period (Thornton et al., 2014). At supra-decadal timescales, climate models underestimated regional but not global temperature variability. Proxy reconstructions and model simulations are essential for detecting multidecadal to centennial climate fluctuations (Laepple et al., 2023). Climate models and proxy interpretations are inconsistently less reliable on multidecadal and longer timescales than proxy-based reconstructions, implying deficiencies. Studies found, in contrast, that simulated and proxy reconstructed temperature variations were in agreement. Climate models simulating past climate states cannot replicate regional climate variations for longer timescales, thereby underestimating regional variability on multidecadal and longer timescales and biasing climate projections and attribution studies (Laepple et al., 2023).

Multiple factors contribute to climate variability. Internal variability and external forcing affect rainfall in the southwestern Australian region. During 2001–2020, southwestern Australia’s cool-season rainfall declined by 20% compared to the 1901–1960 average. Models simulate realistic levels of decadal variability, suggesting that a large proportion is underestimated and external forcing contribution is significant (Rauniyar et al., 2023).

The pH and temperature of seawater in reef environments vary significantly from day to day. Cycles of variations occurred within 24 h, associated with the daily light cycle, and then within 12 h, associated with the semi-diurnal tides of the atoll. The PH, temperature, and carbonate chemistry were monitored continuously and periodically to determine the variability, showing that seawater temperature did not determine pH variability, and the relative balance of net organic carbon metabolism modulates carbonate chemistry on the atoll throughout the day (de Almeida et al., 2023).

The data exemplify the CDP, in which the range within which the variability occurs is related to internal and external variables.

#### 1.4.2 Per the CDP, variability underlines human disparity

Cell-to-cell variability has stochastic and deterministic components, but defining, quantifying, and disentangling them is challenging (Brückner et al., 2020). Single-cell technology enables quantifying variability from cell to cell in various biological contexts. The extrinsic noise can result from non-observable changes in cellular components such as cell cycle, cell-to-cell signaling, and metabolism within an otherwise homogeneous population (Arriaga, 2008). Nevertheless, it is unknown whether these sources of extrinsic noise are independent of each other or whether they are stochastic or deterministic. Cells employ a variety of regulatory mechanisms to buffer variations in noise, resulting in lower levels of noise in a population as a whole (Eling et al., 2019; Baudrimont et al., 2019).

When multiple tissues or individuals are compared, biological noise is created (Eling et al., 2019). When omic data is sequenced from the whole cell or the nucleus, it produces biological noise. Biological noise increases with the difference between tissues, nucleus or entire cell sequencing, and data obtained from different individuals (Janssen et al., 2023). Datasets must preserve biological meaning while removing the batch effect, such as preserving highly variable proteins so that downstream analysis can be more accurate (Koca and Sevilgen, 2024).

DNA loops, chromatin domains, and higher-order compartments contribute to the complex three-dimensional organization of genomes in the cell nucleus. These features are present in most types of cells and tissues. Transcription is stochastic, and chromatin architecture varies widely among cells. A cell- and allele-specific variability in genome architecture reflects intrinsic and extrinsic sources of variability, leading to structural heterogeneity in genome function (Finn and Misteli, 2019).

Submaximal exercise capacity, for example, indicates cardiorespiratory fitness. A heritability level of about 40% characterizes submaximal exercise capacity. Physical working capacity (PWC150) was used to identify panels of genes associated with human variability in intrinsic PWC150 (iPWC150) and its trainability (dPWC150). By combining genome-wide association and skeletal muscle gene expression with plasma proteomics and metabolomics experiments, genes, proteins, and metabolites were associated with iPWC150 or dPWC150. Numerous muscle and cardiovascular phenotypes have been associated with DNA variants. Two panels of prioritized 13 genes and pathways of biological relevance to iPWC150 and dPWC150 were identified, suggesting that iPWC150 involves genes and pathways different from those of dPWC150. Data indicate that skeletal muscle morphology, metabolism, and red blood cell oxygen-carrying capacity affect submaximal exercise capacity and support the linkage between variability and genetic phenotypes (Hota et al., 2023).

#### 1.4.3 In the CDP, randomness is exploited to prevent the accumulation of mutational harm

“Organelle” DNA, or oDNA, contains instructions for building blocks that can be changed. Some leaves lose their ability to photosynthesize when they become bleached (Broz et al., 2023). By exploiting randomness, plants prevent mutational damage from accruing over time. The mutations inherited from a mother are transmitted to the offspring, resulting in a plant’s descendants dying off. Plants instead spread out the harm they inherit so that while some offspring receive many mutations, others receive fewer. In both animals and humans, this process is called segregation. It relies on plants producing random variances in their offspring. The inherited damage of plants is spread randomly. During cell division, oDNA molecules are randomly distributed, and some of the oDNA molecules are overwritten with others, causing plant segregation over time and between mothers and daughters. By sorting different types of oDNA into different germline cells, selection against damaged oDNA is partially mediated. There is a gradual but continuous segregation process during the development of plants, with a more rapid increase between the inflorescence formation and the next-generation of leaves (Broz et al., 2023). Per the CDP, the data exemplifies the importance of genetic variability for proper system functionality.

#### 1.4.4 According to the CDP, systems malfunction is a result of inappropriate variability

Due to the stochastic nature of biochemical reactions, proteins and mRNAs are produced differently within cells (Soltani et al., 2016). Typically, this source of variation is termed “noise”. Multiple processes contribute to phenotypic variability by amplifying and attenuating noise (Hansen and Weinberger, 2019). Per the CDP, the inherent variability is mandatory for proper functionality, and malfunctions are linked with out-of-range variability. It reflects the dysfunction of the boundaries of the variability, which cannot be compatible with the environmental noise.

*Escherichia coli*’s chemotaxis network modulates its random swimming pattern to navigate its environment. It must deal with unavoidable fluctuations in the number of its molecular constituents. The chemotaxis network’s output is measured by the probability of clockwise rotation (CW), or CW bias, whose temporal fluctuations proxy network noise. A quantification of fluctuations in chemotaxis signaling is achieved by observing the switching statistics of flagella in optically trapped *E. coli* cells over the dynamic range of a network. A steady CW bias fluctuation is mandatory for driving flagellar switching and cell tumbling. Chemical stimulation causes a decrease in the network changes. Based on gene expression noise research, a stochastic theory demonstrated bursts that drive CW bias fluctuations. CW bias is constrained by an intrinsic kinetic ceiling on network activity, suppressing fluctuations. A steep gradient may also be prevented from tumbling unproductively by this limit (Bano et al., 2023). It exemplifies a CDP-based range of the inherent system variability.

Neurons respond to stimuli in various ways, including time-dependent and independent inputs or post-synaptic potentials resulting from interactions between them. Incoming stimuli resemble noise or a random variable. Gaussian white noise is considered a standard function for a stochastic model of neurons. The transmission of signals is affected by pulse width. By examining the role of noise and finite pulses, neurons show fluctuating interspike intervals. Once synchronous dynamics reach some critical value, they collapse into asynchronous dynamics. As a result of such abrupt changes in dynamics, synchronous and asynchronous firing behavior coexist (Pimenova et al., 2016). The data exemplifies the importance of a range of variability supporting the B = F formula of the CDP.

When healthy, biological and physiological systems display complex randomness with specific characteristics that are scale-invariant and nonlinear. A breakdown in these characteristics results in inappropriate variability linked to pathological states and conditions. Research on cardiac dynamics, locomotion, and brain function supports these concepts (Peng et al., 1995; Ivanov et al., 1996; Ivanov et al., 1999a; Ivanov et al., 1999b; Kantelhardt et al., 2002; Schumann et al., 2010; Yang et al., 2011). Physiological variability is vital for maintaining brain dynamics at critical levels and for proper brain function during sleep, wakefulness, and cognitive processes. Previous studies have shown that neuronal noise in human and animal brains is vital to sleep arousal. This neuronal noise also influences adverse clinical events, such as sudden infant death syndrome, linked to reduced variation in neuronal noise (Dvir et al., 2018). These earlier basic research studies laid the foundation for modern clinical practice.

Metabolic syndrome is associated with physiological variability in mitochondrial rRNA (Pecina et al., 2023). Symptoms of metabolic syndrome are related to common mitochondrial DNA sequence variants. High-fat diets affected the metabolic phenotype of rat strains with identical nuclear but unique mitochondrial genomes. Insulin resistance development is related to mitochondrial rRNA sequence variation due to the accumulation of diacylglycerol due to tissue-specific reductions in oxidative capacity. The variation in the 12 S rRNA sequence affects the assembly and translation of mitochondrial ribosomes, causing metabolic disturbances (Pecina et al., 2023). It reflects the importance of a personalized variability signature in the subject’s phenotype.

A persistent fluctuation in blood glucose levels is referred to as glycemic variability and is linked to anxiety and depression. To potentially improve the effects of diabetes, glycemic control, and fluctuations must be maintained at an optimal level in the comprehensive management of diabetes (Shi et al., 2023). A measure of glucose variability is the coefficient of variation (CV), calculated by dividing the standard deviation by the mean blood glucose level. Glycemic variability is associated with severe consciousness disturbance and in-hospital mortality in critically ill patients with cerebrovascular disease (CVD) with cerebral infarction and non-traumatic cerebral hemorrhage. A log-transformed CV was associated with cognitive impairment and in-hospital mortality. The data suggested that enhancing glycemic variability stability may reduce adverse outcomes in patients with severe CVD and supports the CDP concept that variability needs to be constrained for proper function (Cai et al., 2023). The data exemplifies the linkage of out-of-range variability with organ malfunction.

Central aponeurosis (CA) cross-sectional non-uniformities were evaluated as a marker for the central region of the rectus femoris (RF) in patients with low physical activity-induced muscle atrophy. Muscle thickness was thinner at the edge than at the center, indicating a non-uniform morphology in the RF. There is a wide variation in the cross-sectional area of atrophic muscle and a non-uniformity in its cross-sectional area that can identify the RF’s center, supporting the role of variability range in diseased conditions (Takahashi and Okura, 2023).

Data from individuals who underwent detailed hormonal sampling were used to quantify reproductive hormone variability due to pulsatile secretion, diurnal variation, and feeding based on CV and entropy. One reproductive hormone measure could quantify reproductive hormone variability due to pulsatile secretion, diurnal variation, and nutrient intake. Based on the data, the reliability of reproductive hormone measurements can be evaluated by quantifying the variability of a single measurement (Abbara et al., 2023). Adrenocorticotropic hormone (ACTH) levels before and after transsphenoidal surgery (TSS) indicate remission. Using single ACTH measurements to determine Cushing’s disease (CD) remission is challenging. Variability suppression of ACTH was observed in CD, with remission associated with restored variability, suggesting that the degree of variability can serve for disease prediction (Alvarez et al., 2023).

All-cause dementia and neurodegenerative conditions are associated with sleep disturbances and clinical sleep disorders. Sleep variability over a long period is significantly associated with cognitive impairment, indicating that sleep duration instability may play a role in cognitive decline (Keil et al., 2023).

According to the CDP, these examples illustrate that diseases are associated with inappropriate variability borders, emphasizing the need for adequately functioning borders that adapt to dynamic changes in the environment to maintain an adequate range of variability in a system.

#### 1.4.5 Per the CDP, the environment influences physiological variability, and species interactions influence eco-evolutionary outcomes

The variability of healthy biological rhythms exhibits fractal-like patterns, while pathology and disease degrade them (Rhea et al., 2014; Sturmberg et al., 2015). Biological rhythm patterns may interact to reflect overall health or lack of it, depending on how they interact (Zueva, 2015).

Human gait variability is essential for safe and adaptive gait and interacting effectively with dynamic environments. Health gait variability exhibits fractal-like patterns, indicating optimal connectivity between biological processes. The breakdown of these patterns suggests a decrease in the interaction between system elements (Vaz et al., 2020). Previous studies have reported the discovery of scale-invariant structures, fractal patterns, self-similarity, and power-law long-range correlations observed across multiple measurement scales in gait dynamics, wrist motion, and locomotor control (Hausdorff et al., 2001; Hu et al., 2004; Ashkenazy et al., 2002; Ivanov et al., 2007). These pioneering works demonstrated that, although stochastic, the variability in physiological systems is not random but adheres to specific dynamical laws that vary with physiological states and pathological conditions. Among the features of gait variability that distinguish functional from dysfunctional locomotor systems is the temporal structure of stride-to-stride fluctuations. These fluctuations in healthy and functional gait exhibit fractal-like patterns and are self-similar across multiple measurement scales (Bock and Beurskens, 2010; Phinyomark et al., 2020). There is a significant, robust, positive correlation between the temporal structure of gait and the patterns of muscle activity in older adults. During walking, muscle activity variability patterns and stride-to-stride fluctuations of older adults are positively correlated, and biological rhythms that make up the human neuromuscular system are interconnected (Jordão et al., 2023). The data supports the importance of variability in adapting to environmental pressures.

Heart rate variability (HRV) refers to the difference in time between pulses, which reflects the effects of the autonomic nervous system (ANS). When a subject is relaxed, the HRV tends to vary, but when they are stressed, it becomes more regular. HRV dampens with age, with more regular intervals between heartbeats. As a result, the adaptive response is more rigid—stimulating the vagus results in diminished release of inflammatory cytokines (Chen P. C. et al., 2021). Previous studies have highlighted the occurrence of self-similarity across various measurement scales, as evidenced by heart rate variability (HRV) (Ivanov et al., 2001; Schulte-Frohlinde et al., 2001; Nunes Amaral et al., 2001; Ashkenazy et al., 2001; Ivanov et al., 2004; Ivanov, 2007). In patients with Type II diabetes who received seasonal flu vaccinations, CRP, a marker for inflammation, was transiently elevated, while HRV declined simultaneously (Lanza et al., 2011). Post-COVID vaccination, HRV decreased significantly on day two and returned to baseline by day 10 (Kerkutluoglu et al., 2023). When humans hear or read grammatical mistakes, they experience a physiological stress response, and their HRV is more regular. When subjects heard speech samples without grammatical errors, their HRV was more variable, indicating they were more relaxed, and the HRV became more regular when grammatical errors were heard (Divjak et al., 2024). The data reflects the CDP, which measures the inherent variability in response to environmental pressures.

Herbivory is a selection pressure on plants reflected in physical and chemical adaptations that prevent animals from eating their tissues. Most ecosystems rely heavily on interactions between plants and herbivores, but their strength is highly variable. Plant-herbivore biology is influenced by the variability within a system, from ecological stability to plant defense evolution. The variability in herbivory within populations increases with latitude, decreases with plant size, and is phylogenetically structured. There is a weak increase in mean herbivory at lower latitudes and a decrease in variation between individuals. Plant species with smaller leaves showed higher herbivory variability, indicating phylogenetic relationships. Per the CDP, it exemplifies how variability varies across macroscale gradients (Robinson et al., 2023).

### 1.5 Variability-based CDP algorithms

Algorithms that use randomness are a significant challenge (Flanigan and Procaccia, 2023). Numerous factors make real-world data noisy and imperfect, resulting in unpredictable outcomes (Hariri et al., 2019).

Information transmission in biological signaling circuits is described as a noise filter. Cells need accurate, real-time data about their environment to propagate, amplify, and process signals. However, biochemical reaction networks cannot provide accurate representations of the data. In addition to filtering the noise, biology must predict the environment’s current state based on delayed information caused by the finite speed of chemical signaling (Tsimring, 2014; Zechner et al., 2016). The biochemical noise filter is derived from studies that relate signaling fidelity in cellular circuits to optimal noise filtering. The framework provides a versatile means of determining the maximum mutual information between an environmental signal and a real-time estimate derived from the system. A biological network’s structure and its components’ response times affect the accuracy of that estimate. By tuning enzyme kinetic parameters and populations, evolution may have optimized information transfer (Hathcock et al., 2016).

A fundamental principle in this field is the “Maximum Entropy Principle,” which indicates that the distribution with the highest entropy should be chosen as the least biased model among all probability distributions that meet the specified constraints (Ahmed et al., 2023). This principle is fundamental to many machine learning and deep learning applications. In classification tasks, deep learning models often utilize the Softmax activation function in the output layer due to the principle of maximum entropy (Ahmed et al., 2023; Lin et al., 2016).

Per the CDP, algorithms that account for variability and uncertainty can improve the accuracy of their outputs. Below are several examples of using randomness to improve algorithm accuracy.

#### 1.5.1 Entropy is used in machine learning and information theory

Entropy is associated with the concept of disorder and measures the level of chaos in a dataset. Low entropy values indicate a more predictable and structured dataset, while high entropy values indicate high uncertainty and randomness (Santana-Carrillo et al., 2023). A CDP-based algorithm can be implemented using entropy to improve models’ accuracy by bringing them closer to reality. In information theory and machine learning, entropy measures uncertainty or randomness in a dataset, providing insight into the information or disorder in a system (Author Anonymous, 2023). Information theory defines entropy as the amount of information required to encode symbols (Naeem et al., 2023). Determining the minimum number of bits required to encode each symbol from a source measures the uncertainty or randomness of the source’s output. Whenever all outcomes in a source have equal probabilities, the entropy value is at its maximum. As a result, every outcome has an equal chance of occurring, resulting in the highest level of uncertainty. When there is a high probability of one outcome occurring, entropy is minimized, resulting in low uncertainty (Brisson et al., 2023).

In machine learning, entropy measures the purity or impurity of the data. By evaluating the unpredictability or randomness within a dataset, machine learning optimizes decision-making models (Sarker, 2021). An algorithm can make informed decisions and improve its performance based on a dataset’s level of uncertainty or impurity. It can describe or predict the outcomes of a system by quantifying the level of disorder or randomness in the data (Ali et al., 2022).

Entropy plays a role in supervised learning tasks that learn a mapping between input features and output labels and is used by random forests and decision tree algorithms (Sarker, 2021). Based on entropy, decision trees can determine which attribute or feature reduces entropy most after a split, resulting in more homogeneous subsets and better class separation (Zhang and Gionis, 2023). Decision tree algorithms evaluate the information gain or reduction in uncertainty in datasets by calculating their entropy before and after possible splits (Podgorelec et al., 2009). Decision tree nodes are measured by their entropy, which measures their impurity. Tree-building is stopped when the desired purity or minimum entropy level is reached. Nodes are considered pure if all their examples belong to the same class, resulting in zero entropy and no further splitting required (Zhang and Gionis, 2023). Random forests, which combine multiple decision trees to make predictions and reduce overfitting, use entropy to reduce overfitting. A random forest’s tree-building process is guided by entropy, which calculates the information gained at each split point (Gao et al., 2022). Entropy is used to evaluate the distribution of different classes within a dataset to determine the predictive power of features (Birant and Birant, 2023; Juszczuk et al., 2021; Tsang et al., 2011; Gao et al., 2022; Sun et al., 2019; Abdar et al., 2021; Bezbochina et al., 2023).

#### 1.5.2 Machine learning using CDP-based probabilistic methods

Machine learning models use deterministic approaches, where one outcome can be derived from a set of inputs (Sarker, 2021). This approach does not consider real-world data for its inherent uncertainty and variability. As deterministic models ignore uncertainty, they fail to capture potential outcomes’ ranges and associated probabilities. The result may be overconfident predictions and unreliable decisions (Abdar et al., 2021). The deterministic approach is unable to explain underlying processes or causality. Factors contributing to the outcome are not considered when examining the input-output relationship, which is complex and affected by hidden variables (Karimi et al., 2022). The deterministic model does not generalize well to new and unknown data when it ignores this complexity and lacks flexibility and adaptability. For deterministic models to make predictions, data must be complete and precise and have a fixed relationship between variables. Real-world scenarios, however, are likely to have missing or noisy data, and real-world decision-making involves making choices under uncertain conditions (Gurung et al., 2020; Taghavifard et al., 2009).

Several factors contribute to uncertainty, including incomplete information, noise in the data, and inherent system variability. Probabilistic approaches to machine learning effectively address uncertainty in decision-making (Adnan et al., 2023). Probabilistic models assign probabilities to different outcomes or predictions, indicating their degree of confidence and facilitating the handling of noisy or missing data (Aizpurua et al., 2022; Manzoni et al., 2023). Multiple outcomes or predictions are generated based on the same input, and probabilities are assigned to the different outcomes. The goal of a probabilistic model is not to find a single “correct” answer but to estimate the likelihood of various outcomes and provide a distribution of probabilities associated with each outcome. It provides a more realistic and comprehensive picture of problems because uncertainty is incorporated. It enables more nuanced decision-making, accounting for the various possible outcomes and likelihood (Khodabakhshian et al., 2023). This approach is beneficial when multiple factors contribute to the outcome and there is limited or noisy data to analyze.

An uncertainty in machine learning can be defined as a lack of complete knowledge or a variable outcome and is divided into two types. The aleatoric uncertainty of data stems from its inherent randomness, and the epistemic uncertainty is a lack of knowledge or information about the underlying process (Hüllermeier and Waegeman, 2021). In probabilistic models, the uncertainty associated with a prediction is estimated to get a more comprehensive picture of the underlying data distribution (Enderle et al., 2023).

A probabilistic model is built using various methods, including Bayesian networks, Gaussian processes, and hidden Markov models. Considering uncertainty and variability, these models offer a more robust and flexible framework for analyzing and understanding complex phenomena (Mowbray et al., 2022). A Bayesian network is a model where nodes represent variables, and probabilistic relationships are defined by conditional probabilities (Vaniš et al., 2023). Comparing the complexity and fit of different models is the primary method of Bayesian model selection, which avoids overfitting and selects the model that best represents the underlying process (Piironen and Vehtari, 2017).

In probabilistic modeling, Bayesian inference enables updating beliefs about uncertain quantities based on observed data. It quantifies uncertainty and makes coherent and principled predictions (Etz and Vandekerckhove, 2018). Using observed data describes how to update prior beliefs and probabilities. In a posterior probability model, the evidence in the data is considered along with the prior beliefs to determine the updated knowledge (Liu et al., 2023). This framework combines prior beliefs with data-driven evidence to produce more reliable predictions. Based on Bayesian inference, a posterior distribution describes the range of possible values and their probabilities. By capturing the uncertainty inherent in the data, this distribution allows for more informed decisions to be made (Liu et al., 2023).

It is possible to capture uncertainty using non-parametric methods, like Gaussian processes, that model the underlying distribution. It offers flexibility and estimates uncertainty for predictions suitable for small or noisy data sets (Dudek and Baranowski, 2022). It is possible to estimate uncertainty more robustly by combining multiple models’ predictions using ensemble methods, such as random forests or bagging. By introducing randomness into the learning process, these methods generate models that capture both aleatoric and epistemic uncertainty (Wang et al., 2023). The Monte Carlo method of simulation and the Sequential Monte Carlo method of distribution estimation are often used together to simulate possible outcomes and estimate the distribution of decision outcomes (Endo et al., 2019).

There are challenges to the use of probabilistic approaches (Kobayashi and Hiraishi, 2014). To make reliable predictions, they need substantial data to estimate the underlying uncertainty accurately. Low-quality or inadequate data can lead to unreliable estimates and biased results. Using complex models and large datasets can be computationally demanding. Some probabilistic models cannot scale up as the problem’s complexity and dataset’s size increase (Ding and Bouvry, 2014; Li et al., 2020). Bayesian models are challenging in specifying and communicating probabilistic results to stakeholders and decision-makers (Gao et al., 2022; Sun et al., 2019).

#### 1.5.3 Use of bootstrapping in machine learning

The bootstrapping technique in machine learning comprises resampling with replacement to generate multiple training datasets rather than relying solely on a few observations (Tsamardinos et al., 2018; Huang and Huang, 2023; Mohammed and Kora, 2023). It involves randomly selected observations from the original dataset to create a new training dataset. Every observation has an equal chance of selection, and once selected, it is added back to the original dataset, allowing for duplicate observations and exclusions. A machine-learning model is then trained on the newly generated dataset. A decision tree, random forest, or support vector machine could be used. This model generates predictions for unseen data based on the variability in the bootstrapped dataset. By randomly sampling observations from an original dataset, bootstrapping creates new training datasets iteratively, enabling the estimation of a model’s prediction stability and uncertainty. The size of each new dataset is the same as the original, but some observations may be repeated while others are left out. Due to this sampling bias, the underlying data may appear skewed and affect the model’s generalizability, and without careful management, it may lead to overfitting (Tsamardinos et al., 2018; Iba et al., 2021).

Multiple datasets are generated to simulate the complexity and variability of real-world data (Salman et al., 2021). It improves the models’ stability and uncertainty. When trained on different data variations, models make more accurate predictions by considering possible variations in the underlying data. The method is proper when a limited number of datasets are available (Ahmed et al., 2023; Millard and Richardson, 2015; Chen T. R et al., 2021; Michelucci and Venturini, 2021). Combining predictions from multiple models improves overall performance and reduces overfitting. The variability and uncertainty in machine learning models can be distinct using it. Considering the range of possible outcomes facilitates decision-making by quantifying the uncertainty associated with the predictions (Rao, 2000; de Zarzà et al., 2023).

These methods exemplify the CDP-based models in algorithms for improving data analysis by incorporating the inherent variability of complex systems.

#### 1.5.4 Predictions are improved by using variability in the CDP

The CDP enables improving prediction by accounting for systems’ variability. In intensive care units (ICUs), predicting in-hospital cardiac arrest allows for prompt interventions to improve patient outcomes. The prediction is improved by a machine learning-based real-time model based on HRV measures (Lee et al., 2023). Similarly, intra-individual cognitive variability is a measure of fluctuations in cognitive performance. The variability of thinking, memory, and other behaviors is associated with neurodegenerative disorders such as Alzheimer’s (Bangen et al., 2019; Halliday et al., 2018).

Functional connectivity (FC) is derived from functional magnetic resonance imaging and is a “fingerprint” of cognitive performance and mental health. FC varies significantly across scans and contains biological information. The functional network connectivity (FNC) was extracted using cross-scan FC stability analysis, demonstrating an ability to identify a child from a large group. Cross-scan FNC stability predicted children’s behavior, with higher stability associated with better cognitive performance, longer sleep duration, and less psychotic expression. Children’s connectivity profiles are intrinsically variable and exhibit reliable variability regardless of brain development. It is helpful to use cross-scan connectivity stability to draw inferences about a child’s cognitive and psychiatric development (Fu et al., 2023).

It is common for cardiac disease to affect the heart non-uniformly. Among these characteristics are focal septal or apical hypertrophy with reduced strain in hypertrophic cardiomyopathy, replacement fibrosis with akinesia in infarcted coronary arteries, and a pattern of scarring in dilated cardiomyopathy. Cardiovascular magnetic resonance imaging (CMR)-derived heterogeneity biomarkers facilitate earlier diagnosis, better risk stratification, and more accurate treatment prediction. There are two heterogeneity measures: the mean absolute deviation of the regional standard deviation on native T1 and T2 and the standard deviation of the time to maximum regional radial wall movement (Hesse et al., 2023). The results show how out-of-range variability can serve for the early detection of diseases.

Continuous and dynamic fluctuations in blood pressure levels, blood pressure variability (BPV) refers to the standard deviation of repeated measurements, the coefficient of variation (standard deviation divided by the mean), and metrics considering changes from measurement to measurement. There is a link between BPV and stroke patients’ outcomes. In acute stroke patients without thrombolysis, elevated systolic BPV is associated with poorer outcomes, such as functional disability, mortality, and neurological deterioration before stroke recurrence. A higher risk of mortality and functional disability is associated with higher diastolic blood pressure variability (Chen et al., 2023).

Overall and central obesity burdens and variability are associated with higher all-cause and cardiovascular mortality among men. Women with general obesity and cumulative and variable central adiposity have a higher risk of all-cause mortality (Kohansal et al., 2023).

Based on these data, predictions can be improved by using CDP-based variability algorithms. A digital twin is a virtual representation of a physical object. Including variability in CDP-based digital tween models improves their prediction accuracy in complex systems and enables better monitoring and management of malfunctioning systems (Sigawi and Ilan, 2023).

#### 1.5.5 Improve the efficiency of systems by using CDP-based variability

Based on the CDP, systems malfunction results from an inadequate variability caused by borders that fail to respond adequately to environmental perturbations. Functionality can be improved by regulating the degree of variability (Ilan, 2022a; Ilan, 2023b).

The concept of the CDP was introduced in earlier studies, which showed that physiological variability occurs within a specific range known as the “critical zone.” Beyond this zone, the scale-invariant and nonlinear patterns of physiological variability begin to break down, leading to system dysfunction. In the case of heart rate variability (HRV), it has been shown that emergencies in this critical zone stem from regulatory mechanisms of the central nervous system (Ivanov et al., 1998). It is suggested that this zone is crucial for health, as driving variability out of the critical zone results in clinical perturbations.

Light therapy improves sleep quality and inter-daily stability, which measures the strength of circadian rhythms, thereby reducing intraday variability and measuring how often someone transitions between rest and activity. There is an improvement in symptoms associated with Alzheimer’s disease after using light therapy (Zang et al., 2023). The data support the CDP-based concept that constraining variability can improve functionality.

The digital pill improves response to medications and medical devices using CDP-based algorithms by incorporating variability into protocols (Ilan, 2021a). CDP-based second-generation AI algorithms have three levels designed to improve responsiveness, overcome tolerance to therapies, and prevent partial or complete loss of effectiveness of chronic therapies (Ilan, 2021a; Ilan, 2020c; Ilan, 2020d; Ilan, 2022d; Hurvitz and Ilan, 2023). Similarly, chronicity and repeatability are associated with loss of functionality in other areas of life, such as work burnouts and sports activity (Gelman et al., 2022). In the first level of the algorithm, an open-loop platform, variability is introduced into interventions without regard to outcome. The range of variability is predefined by the user or the system manager (Sigawi et al., 2023; Gelman et al., 2023).

In the second level, a closed-loop system regulates the variability based on the outcome. Algorithms are designed to reach predefined outcomes and continuously adapt to reduce or increase the variability to maintain a high degree of functionality under changing conditions. The degree of variability is tailored to a target person or system and personalizes the algorithm output. As a dynamic platform, it constantly adapts to internal and external changes in each subject or target system (Ilan, 2021a; Ilan, 2020d; Ilan, 2022d; Hurvitz and Ilan, 2023).

At the third level, the algorithm receives additional inputs from quantifying the variables in the target system (e.g., HRV, BPV). The algorithm regulates the degree of variability based on these dynamic inputs. The third level enables a sophistication of the output variability by bringing it closer to the dynamic inherent variability of a system. CDP-based second-generation AI systems provide a platform for improving the functionality and efficiency of all systems of nature by using personalized variability signitures (Gelman et al., 2022; Kessler et al., 2020; Ishay et al., 2021a; Kolben et al., 2021; Kenig et al., 2021; Azmanov et al., 2021; Potruch et al., 2020; Isahy and Ilan, 2021; Khoury and Ilan, 2019; Khoury and Ilan, 2021; Kenig and Ilan, 2019; Ilan, 2019f; Gelman et al., 2020; Ishay et al., 2021b; Ilan and Spigelman, 2020; Hurvitz et al., 2021; Ilan, 2021b; Azmanov et al., 2022; Hurvitz et al., 2022; Kolben et al., 2023; Adar et al., 2023; Ilan et al., 2019).

### 1.6 The CDP defines human behavior as being driven by randomness

Including random processes in decision-making avoids the inevitable repetition inherent in human-made decisions. Mixing randomness with predictability is known as a “mixed strategy.” (Randomness to optimize your decision, 2023).

London Underground strike in February 2014 helped commuters find better routes. As a result of the strike, many commuters were forced to experiment with alternative routes randomly, and 5% adopted them permanently. Many commuters failed to modify their work journey to find the optimal commute. They settled for a passable trip to avoid the risk of extended commutes that might result from randomly experimenting with their journeys. The strike’s randomizing effect led some commuters to find better routes in the long run (Rauch and Larcom, 2015).

When faced with an excessive choice, randomness can solve “analysis paralysis.” (Randomness to optimize your decision, 2023). As a result of consumers’ freedom of choice, a competitive environment is supposedly created, driving innovation and efficiency and improving the overall consumer experience. Randomly minimizing the choices can lead to a better result. Fewer choices may seem counterintuitive, but it can be beneficial since fewer choices translate into higher sales (Randomness to optimize your decision, 2023). Consumers can experience anxiety from the fear of missing out (FOMO) on a better opportunity to the loss of presence in a chosen activity and regret from making the wrong choice (Kim et al., 2020; Alfina et al., 2023). Consumers can feel that no experience is genuinely satisfactory with so many choices and “analysis paralysis.” According to the paradox of choice, the greater the options, the less likely potential customers are to complete a purchase (Randomness to optimize your decision, 2023; Karlık, 2020; Khan et al., 2022; Kinjo and Ebina, 2015).

Wealth inequality may reflect “pure randomness.” When people are wealthy, it is because they are lucky, a result of randomness. Even if everyone followed the same rules and had equal talent, a string of chance events determined the ultimate distribution of wealth in society (Boghosian, 2023; Li et al., 2019). Wealth distribution arises from complex stochastic processes, but empirical evidence shows that it follows universal power laws. These laws describe the distribution of wealth among individuals, companies, and countries, indicating that wealth distribution is not random. This phenomenon is related to the Matthew effect, which suggests that the rich tend to get richer (Coelho et al., 2008). Wealth accumulation at the personal, corporate, or national level is stochastic; however, it is not random (Terwijn, 2016; Li et al., 2016; Flanigan et al., 2021; Abrahams et al., 2023; Vossen and Hofmans, 2021).

Another example is choosing a panel that can represent a population. In mini-public selection methods, participants are selected randomly rather than based on filters or personal connections (Stone et al., 2023). The definition and exploration of the design space of randomness may help determine which properties of randomness support legitimacy (Terwijn, 2016). The challenge is quantifying this randomness’s conceptual normative properties and determining which properties can be achieved by efficient algorithms. The design space of randomness in a selection algorithm must be defined to determine what randomness remains available after imposing representation constraints (Li et al., 2016). Random algorithms distribute the probability across multiple panels. A panel distribution can determine what randomness is available after the quotas are imposed (Flanigan and Procaccia, 2023). Due to this relationship between the panel distribution and individual selection probabilities, the selection algorithm can allocate selection probabilities across pool members. All pool members cannot have equal selection probabilities. It is possible to design the selection algorithm to minimize the disruption caused by envy within the pool (Flanigan et al., 2021). By shaping the likelihood of these groups being well-represented, the available randomness can promote the representation of additional groups in random ways. It is possible to select any measure of closeness to equal selection probabilities using algorithms (Flanigan and Procaccia, 2023). Nevertheless, there is a challenge in ensuring representation through the randomness of selection algorithms (Author Anonymous, 2023).

Individuals’ self- and other-rated personality variability is related to their job performance (Abrahams et al., 2023; Vossen and Hofmans, 2021). Within-person variability, above mean-level personality, positively correlated with self-rated job performance. Within-person variability rated by others was negatively associated with other performance ratings. Interactions with mean-level personality were observed, with less adaptive personalities showing adverse effects of variability and more adaptive personalities showing positive effects. It appears that perceptions of within-person personality variability can influence performance evaluations beyond personality traits, although its desirability varies according to an individual’s personality traits (Abrahams et al., 2023). The data further supports the CDP-based notion that the degree of variability impacts performance in a personalized way.

In summary, the CDP provides a platform that underlies the function of all systems. Per this principle, inherent variability is mandatory for proper function, and system malfunction is an inappropriate functioning of the variability’s boundaries designed to enable adaptability to perturbations. The CDP-based algorithms that implement and regulate the degree of variability in systems can improve their accuracy and predictability and are explored to improve systems’ functionality. Future studies will explore using CDP-based platforms to improve system efficiency and overcome failures.

## Abbreviations

CDP, constrained disorder principle; CV, coefficient of variation; AI, artificial intelligence; HRV, heart rate variability; ANS, autonomic nervous system; BPV, blood pressure variability.
